# Pro-inflammatory mechanisms of muscarinic receptor stimulation in airway smooth muscle

**DOI:** 10.1186/1465-9921-11-130

**Published:** 2010-09-28

**Authors:** Tjitske A Oenema, Saeed Kolahian, Janke E Nanninga, Daniëlle Rieks, Pieter S Hiemstra, Suzanne Zuyderduyn, Andrew J Halayko, Herman Meurs, Reinoud Gosens

**Affiliations:** 1 Department of Molecular Pharmacology, University of Groningen, The Netherlands; 2 Department of Basic Sciences, University of Tabriz, Iran; 3 Department of Pulmonology, Leiden University Medical Center, The Netherlands; 4 Department of Physiology & Internal Medicine, University of Manitoba, Winnipeg, MB, Canada

## Abstract

**Background:**

Acetylcholine, the primary parasympathetic neurotransmitter in the airways, plays an important role in bronchoconstriction and mucus production. Recently, it has been shown that acetylcholine, by acting on muscarinic receptors, is also involved in airway inflammation and remodelling. The mechanism(s) by which muscarinic receptors regulate inflammatory responses are, however, still unknown.

**Methods:**

The present study was aimed at characterizing the effect of muscarinic receptor stimulation on cytokine secretion by human airway smooth muscle cells (hASMc) and to dissect the intracellular signalling mechanisms involved. hASMc expressing functional muscarinic M_2 _and M_3 _receptors were stimulated with the muscarinic receptor agonist methacholine, alone, and in combination with cigarette smoke extract (CSE), TNF-α, PDGF-AB or IL-1β.

**Results:**

Muscarinic receptor stimulation induced modest IL-8 secretion by itself, yet augmented IL-8 secretion in combination with CSE, TNF-α or PDGF-AB, but not with IL-1β. Pretreatment with GF109203X, a protein kinase C (PKC) inhibitor, completely normalized the effect of methacholine on CSE-induced IL-8 secretion, whereas PMA, a PKC activator, mimicked the effects of methacholine, inducing IL-8 secretion and augmenting the effects of CSE. Similar inhibition was observed using inhibitors of IκB-kinase-2 (SC514) and MEK1/2 (U0126), both downstream effectors of PKC. Accordingly, western blot analysis revealed that methacholine augmented the degradation of IκBα and the phosphorylation of ERK1/2 in combination with CSE, but not with IL-1β in hASMc.

**Conclusions:**

We conclude that muscarinic receptors facilitate CSE-induced IL-8 secretion by hASMc via PKC dependent activation of IκBα and ERK1/2. This mechanism could be of importance for COPD patients using anticholinergics.

## Background

Chronic obstructive pulmonary disease (COPD) is an inflammatory lung disease characterized by airflow limitation that is not fully reversible [1]. The pathophysiology of COPD is mainly caused by cigarette smoke. COPD is associated with an increase in local and systemic inflammatory cytokines including TNF-α and IL-1β [2]. Furthermore, clinical studies reported that the levels of IL-8 [3] and leukotriene B_4 _[4] are correlated to the proportion of neutrophils present and are increased in induced sputum of COPD patients. Additionally, during exacerbations periods, IL-8 levels are increased [3]. Attracted by IL-8, neutrophils play a significant role in the pathogenesis of COPD. Neutrophils promote tissue inflammation and injury by inducing the release of mediators including elastase, metalloproteases and reactive oxygen species [4].

Acetylcholine, the primary parasympathetic neurotransmitter in the airways plays an important role in COPD, by regulating bronchoconstriction and mucus production [5]. Parasympathetic tone may be increased in COPD [5]. Therefore, anticholinergics -including tiotropium bromide, a long-acting bronchodilator- are often used as a mainstay therapy for COPD [6]. Recently, however, it has been established that activation of the cholinergic system may also contribute to inflammatory responses in the lung. For example, the release of IL-8 and leukotriene B_4 _by bronchial epithelial cells [7,8] and alveolar macrophages [9]*in vitro *appears to be induced by acetylcholine, resulting in increased neutrophil, monocyte, and eosinophil chemotactic activities, an effect that may be enhanced in COPD. Also, animal studies showed that anticholinergics are capable of reducing neutrophilic and eosinophilic inflammation induced by inhaled diesel-soot [10], inhaled allergen [11], or LPS [12]. Furthermore, it has been reported that airway vascular leakage is mediated by muscarinic receptors [13]. Collectively, these findings suggest a role in pro-inflammatory responses for muscarinic receptors. Nonetheless, it is still undefined what the potential anti-inflammatory effects of muscarinic antagonists are in the lungs of patients with COPD [14], which is in part due to the unknown mechanisms behind the regulation of inflammatory responses by muscarinic receptors.

Human airway smooth muscle (ASM) has been attributed an important role in pro-inflammatory responses in COPD [5]. These cells are capable of expressing and releasing cytokines and growth factors, including IL-6 and IL-8 [15]. Furthermore, it has been reported that ASM cells express cell surface molecules, which can directly interact with immune cells, suggesting an immunomodulatory role of these cells in COPD [16]. Increased pro-inflammatory cytokine release is induced by stimulating human ASM cells (hASMc) with G- protein-coupled receptors, growth factors and extracellular matrix proteins [15,16]. Additionaly, cigarette smoke can evoke inflammatory responses in human hASMc, such as IL-8 secretion [17]. Muscarinic M_2 _and M_3 _receptors, both G-protein-coupled receptors, are expressed in abundance in hASMc, suggesting that acetylcholine regulates inflammatory responses by ASM [18]. Indeed, we recently reported that muscarinic receptor stimulation augments cigarette smoke extract (CSE)-induced IL-8 secretion by hASMc, which was mediated by the muscarinic M_3 _receptor subtype [19].

Although these observations illustrate the potential role for acetylcholine in regulating airway inflammation, the mechanism(s) by which muscarinic receptors regulate inflammatory responses are still unknown. In the present study, we investigated the regulation of cytokine secretion from hASMc by muscarinic receptors, alone and in concerted action with various pro-inflammatory stimuli involved in the pathogenesis of COPD. In addition, we investigated the intracellular signalling mechanisms involved, in particular the role of protein kinase C (PKC) and downstream pathways.

## Methods

### Antibodies and reagents

Methacholine chloride (MCh) was purchased from ICN Biomedicals (Zoetermeer, the Netherlands). GF109203X and U0126 were both from Tocris Cookson Inc. (Bristol, UK). SC514 was obtained from Calbiochem (Amsterdam, The Netherlands). PMA, mouse anti-ß-actin antibody, horseradish peroxidase (HRP)-conjugated rabbit anti-mouse antibody, HRP-conjugated goat anti-rabbit, recombinant human TNF-α, and IL-1β were purchased from Sigma-Aldrich (Zwijndrecht, The Netherlands). Human recombinant platelet-derived growth factor-AB (PDGF-AB) was from Bachem (Weil am Rhein, Germany). Phospho-p44/42 MAPK (ERK1/2) (Thr202/Tyr204) antibody and p44/42 MAPK (ERK1/2) antibody were obtained from Cell Signalling Technology (Beverly, CA, USA). Rabbit anti-IκBα (clone-15) was purchased from Santa Cruz Biotechnology, INC (Santa Cruz CA, USA). All other chemicals were of analytical grade.

### Cell culture

Human bronchial smooth muscle cell lines immortalized by stable expression of human telomerase reverse transcriptase (hTERT) were prepared as described previously [20]. The primary cultured human bronchial smooth muscle cells used to generate these cell lines were prepared from macroscopically healthy segments of 2nd-to-4th generation main bronchus obtained after lung resection surgery from patients with a diagnosis of adenocarcinoma. All procedures were approved by the Human Research Ethics Board of the University of Manitoba. Cells were grown to confluence using DMEM supplemented with 10% FBS, 100 μg/mL streptomycin, 100 U/mL penicillin and 1.5 μg/mL amphotericin B. Cultures were maintained in a humidified incubator at 37°C-5% CO2, and media was changed every 2-3 days.

### Cytokine release

Cells were cultured in 24 well plates and grown until confluence followed by serum-deprivation for 1 day in DMEM supplemented with antibiotics (100 μg/mL streptomycin, 100 U/mL penicillin and 1.5 μg/mL amphotericin B) and ITS (5 μg/mL insulin, 5 μg/mL transferrin, and 5 ng/mL selenium) before each experiment. The cells were stimulated with the muscarinic receptor agonist methacholine chloride (MCh, 10 μM), alone and in combination with either CSE (5%), TNF-α (1 ng/mL), PDGF-AB (30 ng/mL) or IL-1β (1 ng/mL) for 24 hrs to determine cytokine secretion in cell-free supernatant. 100% strength CSE was prepared by combusting two 3R4F research cigarettes (without filter) (University of Kentucky, Kentucky, USA) using a peristaltic pump and passing the smoke through 25 mL of FBS-free medium at the rate of one cigarette per 5 min. CSE was freshly prepared before every experiment and was used within 15 min after preparation. Additionally, where appropriate, hASMc were pre-incubated with either the PKC inhibitor GF109203X (3 μM), the IKK-2-inhibitor SC514 (50 μM) or the MEK inhibitor U0126 (3 μM) for 30 min. Cells were also treated with the PKC activator PMA (0.1 μM). Cytokine levels were quantified using enzyme-linked immunosorbent assays (ELISA), according to the manufacturer's instructions (Sanquin Pharmaceutical services, Amsterdam, The Netherlands). The detection limit was 1 pg/ml for IL-8 and 0.2 pg/ml for IL-6. We diluted samples were needed to remain in the range of the standard curve.

### Preparation of whole cell lysates

HASMc were cultured in 6 well plates and grown until confluence followed by serum-deprivation for 1 day in DMEM supplemented with antibiotics (100 μg/mL streptomycin, 100 U/mL penicillin and 1.5 μg/mL amphotericin B) and ITS before each experiment. The cells were stimulated with the muscarinic receptor agonist MCh (10 μM), alone and in combination with either CSE (5%) or IL-1β (1 ng/mL) for 60 or 120 min. To obtain whole cell lysates, cells were washed once with ice-cold PBS (NaCl 140 mM, KCl 2.6 mM, KH_2_PO_4 _1.4 mM, Na_2_HPO_4_.2H_2_O 8.1 mM, pH 7.4), followed by lysis using ice-cold RIPA buffer (Tris 40 mM, NaCl 150 mM, Igepal 1%, deoxycholic acid 1%, NaF 1 mM, Na_3_VO_4 _1 mM, aprotinin 10 μg/mL, leupeptin 10 μg/mL, pepstatin A 7 μg/mL, β-glycerophosphate 1.08 mg/mL, pH 8.0). Sonicated lysates were assayed for protein content according to Bradford and stored at -20°C until further use.

### Western Blotting

Equal amounts of protein were separated on 10% polyacrylamide-SDS gels and transferred to nitrocellulose membranes. To avoid non-specific binding, membranes were blocked with blocking buffer (Tris-HCl 50 mM, NaCl 150 mM, TWEEN-20 0.1%, non-fat dried milk powder 5%) for 1 hour at room temperature. The membranes were then incubated with specific primary antibodies, all diluted in blocking buffer, for one hour at room temperature. After washing the membranes three times with TBS-T 0.1% (Tris-HCl 50 mM, NaCl 150 mM, TWEEN-20 0.1%) for 10 min, incubation with the secondary antibody conjugated to HRP was performed during 1 h at room temperature, followed by additional three washes with TBS-T 0.1%. Bands were subsequently visualized on film using enhanced chemiluminescence reagents and analyzed by densitometry (Totallab™, Nonlinear dynamics, Newcastle, UK). All bands were normalized to either β-actin for IκBα or to total ERK1/2 for phospho ERK1/2.

### Data analysis

Data are presented as mean values ± SE. Statistical significance of differences between means was determined by a Student's *t*-test or by one-way ANOVA, where appropriate. Data were considered statistically significant when *p *< 0.05.

## Results

### Muscarinic receptor stimulation facilitates cytokine secretion induced by CSE, TNF-α and PDGF-AB

**Figure 1:**
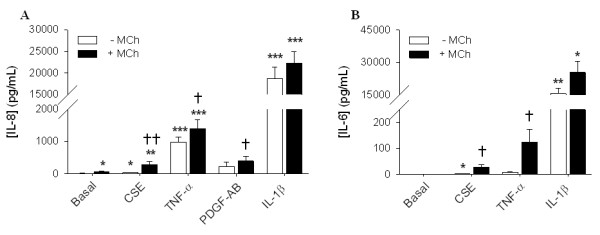
**A-B, Muscarinic receptor stimulation augments cytokine secretion induced by CSE, PDGF-AB and TNF-α, but not by IL-1β**. hASMc were stimulated with CSE (5%, n = 22 and n = 6 for IL-8 and IL-6, respectively), TNF-α (1 ng/mL, n = 17 and n = 5 for IL-8 and IL-6, respectively), IL-1β (1 ng/mL, n = 17 and n = 6 for IL-8 and IL-6, respectively) or PDGF-AB (30 ng/mL, n = 6 for IL-8), in the absence or presence of MCh (10 μM) for 24 hours. Supernatants were analyzed for the presence of IL-8 (A) or IL-6 (B). Data shown are the means ± SE of n independent experiments. *p < 0.05, **p < 0.01 and ***p < 0.001 compared to basal; ^†^*p *< 0.05 and ^††^*p *< 0.01 compared to the absence of MCh (Student's t-test for paired observations).

Recently, it has been reported that stimulation of muscarinic receptors induces the release of IL-8 from human bronchial epithelial cells and facilitates the release of IL-8 from hASMc induced by CSE [8,19]. We evaluated the pro-inflammatory properties of muscarinic receptor stimulation in hASMc, alone and in concerted action with CSE (5%), PDGF-AB (30 ng/mL), TNF-α (1 ng/mL) or IL-1β (1 ng/mL) (Figure 1). Previous findings indicated that the effects of muscarinic receptor stimulation on ASM cytokine secretion were most profound for IL-6 and IL-8 [19], with maximal effects seen at a concentration of 10 μM MCh. Therefore, we used 10 μM MCh and focused on IL-6 and IL-8 cytokines for our measurements. We observed a minor increase in IL-8 induced by MCh alone. CSE alone induced a significant increase of both IL-8 and IL-6 secretion, which was significantly and synergistically amplified by co-stimulation with MCh. In addition, MCh induced a synergistic increase in both IL-8 and IL-6 secretion in combination with TNF-α. Furthermore, a synergistic effect was also observed with the combination of MCh and PDGF-AB for IL-8 secretion. However, the effect of IL-1β, which induced a very high IL-8 and IL-6 production by its own, was not significantly augmented by MCh (Figure 1). IL-8 release in response to IL-1β was found concentration dependent, but treatment with MCh had no additional effects regardless of the concentration IL-1β used (data not shown).

### PKC is involved in the synergistic effect of muscarinic receptor stimulation with CSE

**Figure 2:**
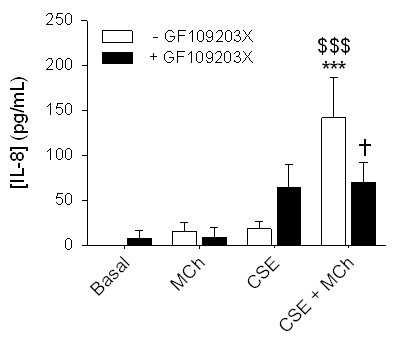
**Involvement of PKC in the potentiation of CSE-induced IL-8 release by muscarinic receptor stimulation**. hASMc were stimulated with CSE (5%) in the absence or presence of MCh (10 μM) and/or GF109203X (3 μM) for 24 hours. Supernatants were analyzed for the presence of IL-8. Data represent means ± SE of 7 independent experiments each performed in duplicate. ***p < 0.001 compared to basal; ^$$$^*p *< 0.001 compared to CSE; ^†^*p *< 0.05 compared to the absence of GF109203X (One-way ANOVA followed by Newman-Keuls multiple comparisons test).

PKC plays an important role as a signalling intermediate in pro-inflammatory cytokine secretion by inducing the activation of several downstream pathways, including the IKK-2/IκBα/NF-κB and Raf-1/MEK/ERK1/2 pathways [21]. The stimulation of muscarinic receptors induces the activation of PKC in ASM [22,23]. We hypothesized therefore, that PKC could play a central role in the synergism between CSE and MCh in IL-8 secretion. HASMc were pretreated with GF109203X (3 μM), a specific PKC inhibitor, and subsequently stimulated with MCh, CSE and their combination (Figure 2). GF109203X significantly inhibited the synergistic effect of MCh on CSE-induced IL-8 secretion, demonstrating a requirement for PKC in this synergism. Remarkably, in the absence of the muscarinic agonist, GF109203X tended to increase the CSE-induced IL-8 secretion.

**Figure 3:**
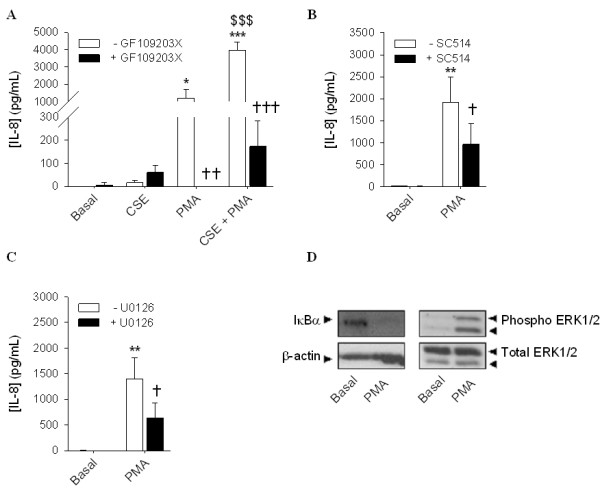
**PKC activation is sufficient to facilitate CSE-induced IL-8 secretion in hASMc**. hASMc were stimulated with PMA (0.1 μM), in the absence or presence of CSE (5%) and GF109203X (3 μM) (Figure 3A), SC514 (50 μM) (Figure 3B) or U0126 (3 μM) (Figure 3C) for 24 hours. Supernatants were analyzed for the presence of IL-8. Data represent means ± SE of 4-6 independent experiments each performed in duplicate. *p < 0.05, **p < 0.01 and ***p < 0.001 compared to basal; ^$$$^*p *< 0.001 compared to PMA; ^†^*p *< 0.05, ^††^*p *< 0.01 and ^†††^*p *< 0.001 compared to the absence of inhibitor (One-way ANOVA followed by Newman-Keuls multiple comparisons test). (Figure 3D) hASMc were stimulated with PMA (0.1 μM) for 1 hour. Cell lysates were analyzed for IκBα breakdown and phosphorylation of ERK1/2 by western blot. β-actin and total ERK1/2 were used as loading controls. Western blots shown are representative of 4 experiments.

To investigate whether PKC activation was sufficient for a synergistic IL-8 secretion in combination with CSE, we used PMA (0.1 μM) as a PKC activator. Indeed, CSE-induced IL-8 secretion was highly augmented in the presence of PMA, which could be abolished to the level of CSE-induced IL-8 secretion when pre-treated with GF109203X (Figure 3A). These data indicate that PKC activation is sufficient for a synergistic interaction with CSE, which is in support of a central role for PKC in regulating the synergy between MCh and CSE. In contrast to MCh, however, PMA induced a considerable IL-8 secretion by itself, which was abolished when the cells were pre-treated with GF109203X.

PKC has been shown to induce activation of the NF-κB and ERK1/2 pathways in different cells [21]. Moreover, it has been reported that the stimulation of muscarinic receptors through acetylcholine mediates the release of IL-8 in human bronchial epithelial cells by NF-κB- and ERK1/2-dependent mechanisms [8]. To test the involvement of the NF-κB and ERK1/2 pathways as a result of PKC activation, hASMc were stimulated with PMA after pre-treatment with either an IKK-2 inhibitor, SC514, or a MEK1/2 inhibitor, U0126. IL-8 secretion induced by PMA was significantly decreased in presence of these pharmacological inhibitors (Figure 3B for SC514 and figure 3C for U0126, respectively). Moreover, western blot analysis indicated that the activation of PKC by PMA induced the phosphorylation of ERK1/2 and the degradation of IκBα in hASMc. Collectively, these data indicate that PKC is able to activate the IκBα/NF-κB and MEK/ERK1/2 pathways, leading to IL-8 secretion from hASMc (Figure 3D).

### Involvement of the IκBα/NF-κB pathway in the synergistic effect of muscarinic receptor stimulation with CSE

**Figure 4:**
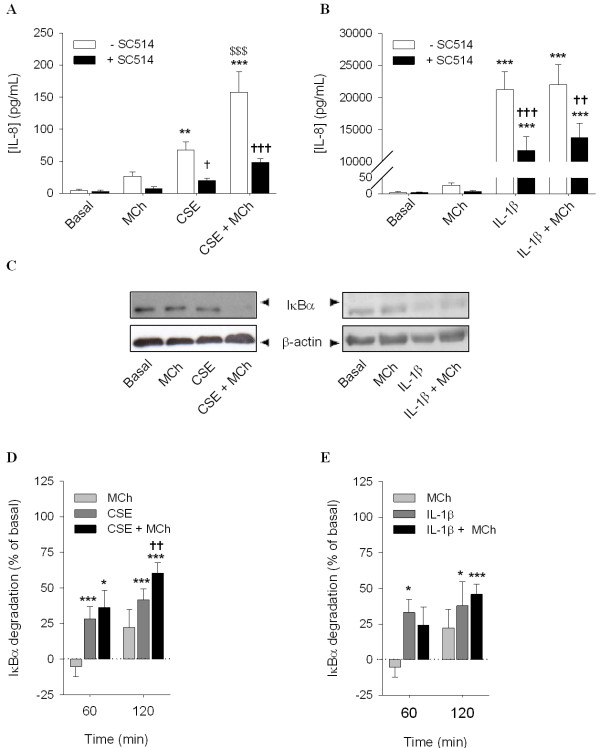
**Involvement of the IκBα/NF-κB pathway in IL-8 secretion induced by CSE, IL-1β and MCh**. hASMc were stimulated with CSE (5%) (Figure 4A) or IL-1β (1 ng/mL) (Figure 4B) in the absence or presence of MCh (10 μM) and/or SC514 (50 μM) for 24 hours. Supernatants were analyzed for the presence of IL-8. Data represent means ± SE of 5 independent experiments each performed in duplicate. **p < 0.01 and ***p < 0.001 compared to basal; ^†^*p *< 0.05, ^††^*p *< 0.01 and ^†††^*p *< 0.001 compared to the absence of SC514; ^$$^*p *< 0.01 compared to CSE (One-way ANOVA followed by Newman-Keuls multiple comparisons test). (Figure 4C, 4 D and 4E) hASMc were stimulated with CSE (5%) (Figure 4C and 4D) or IL-1β (1 ng/mL) (Figure 4C and 4E) in the absence or presence of MCh for 60 min and 120 min (representative blots shown in Figure 4C) as indicated. IκBα degradation was determined by western blot and corrected for the expression of β-actin, which was used as a loading control. Data represent means ± SE of 9-10 experiments. *p < 0.05 and ***p < 0.001 compared to basal and ^††^*p *< 0.01 compared the absence of MCh (Student's *t*-test for paired observations).

HASMc were pretreated with the IKK-2 inhibitor SC514 to test the involvement of this pathway in the synergistic IL-8 secretion by MCh and CSE (Figure 4A). SC514 completely inhibited the MCh- and CSE-induced IL-8 secretion. Furthermore, the synergistic effect of the combination of MCh and CSE was abolished (Figure 4A). These results confirm the involvement of the IκBα/NF-κB pathway in the observed IL-8 secretion. Therefore, we next investigated the effects of muscarinic receptor stimulation on IκBα degradation, alone and in combination with CSE at different time points (60 and 120 min of treatment). IκBα degradation was measured by western blot analysis. Although MCh did not induce significant IκBα degradation by itself, it augmented the response induced by CSE, particularly after 120 min of incubation (Figure 4D). Overall, these results indicate that muscarinic receptor stimulation promotes the activation of the IκBα/NF-κB pathway induced by CSE, which likely contributes to the synergistic IL-8 secretion. Interestingly, and in line with the lack of effect of MCh on IL-1β-induced cytokine secretion, MCh did not augment maximal IL-1β-induced IκBα degradation at t = 60 and 120 min (Figure 4E). However, IL-1β-induced IL-8 secretion in presence or absence of MCh, was significantly inhibited by SC514 (Figure 4B).

### Involvement of the MEK/ERK1/2 pathway in the synergistic effect of muscarinic receptor stimulation with CSE

**Figure 5:**
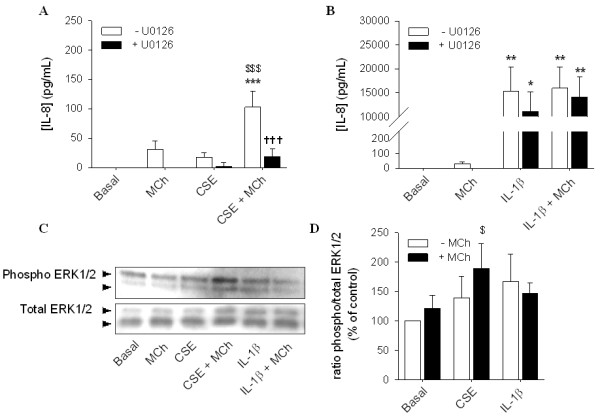
**Involvement of the MEK/ERK1/2 pathway in IL-8 release induced by MCh**. hASMc were stimulated with CSE (5%) (Figure 5A) or IL-1β (1 ng/mL) (Figure 5B), in the absence or presence of MCh (10 μM) and/or U0126 (3 μM) for 24 hours. Supernatants were analyzed for the presence of IL-8. *p < 0.05 and **p < 0.01 compared to basal; ^$$$^*p *< 0.001 compared to CSE alone, ^†††^*p *< 0.001 compared to the absence of U0126. (One-way ANOVA followed by Newman-Keuls multiple comparisons test). (Figure 5C and 5D) hASMc were stimulated with CSE (5%) or IL-1β (1 ng/mL) in the absence or presence of MCh (10 μM) for 60 min. Cell lysates were analyzed for phospho-ERK1/2 by western blot and corrected for the expression of total ERK1/2, which was used as a loading control. Data represent means ± SE of 5 independent experiments each performed in duplicate. ^$^*p *< 0.05 compared to CSE alone (Student's *t*-test for paired observations).

To test the involvement of the MEK/ERK1/2 pathway in IL-8 secretion induced by MCh and CSE, we pretreated the cells with the MEK1/2 inhibitor, U0126 (3 μM) (Figure 5A). In the presence of U0126, IL-8 secretion induced by co-stimulation of CSE with MCh was significantly decreased (Figure 5A). These results confirm the involvement of the MEK/ERK1/2 pathway in the observed IL-8 secretion. Therefore, we next assessed phosphorylation of ERK1/2 induced by MCh and CSE (Figure 5C and 5D). Although, ERK1/2 phosphorylation was not significantly increased when cells were stimulated with MCh alone after one hour of incubation, 15 min incubation is sufficient to induce significant ERK1/2 phosphorylation [23]. In combination with CSE, MCh induced a significant increase in the phosphorylation of ERK1/2 at this time point (one hour). These results support the involvement of the ERK1/2 pathway in the synergism between CSE and MCh at the level of IL-8 secretion. In contrast, IL-1β induced ERK1/2 phosphorylation was not increased by MCh and also pre-treatment with U0126 had no effect (Figure 5B and 5D). These results are in agreement with the results of Orsini, et al., demonstrating that IL-1β can induce a transient phosphorylation of ERK1/2 in human airway smooth muscle cells [24].

## Discussion

In the present study, we demonstrate that muscarinic receptors stimulate the secretion of the pro-inflammatory cytokine IL-8 from hASMc, and augment the response induced by TNF-α, CSE and PDGF-AB. Furthermore, we dissected the underlying mechanism of the synergistic IL-8 production. To permit the release of the pro-inflammatory cytokine IL-8 after activation of the muscarinic receptors and CSE, activation of PKC is required, which is followed by the breakdown of IκBα. In parallel, the activation of PKC leads to the stimulation of MEK1/2 inducing the phosphorylation of ERK1/2. Both pathways regulate IL-8 secretion, which, as previously described, is dependent on NF-κB and AP-1 IL-8 promoter activation [25].

Our current and previously published data [19] indicate that the activation of muscarinic receptors in hASMc facilitates the secretion of the pro-inflammatory cytokines IL-6 and IL-8 in combination with CSE and pro-inflammatory cytokines. Muscarinic receptor stimulation also promoted IL-8 secretion by itself, though only to a relatively minor extent. This suggests that the effects of muscarinic receptor stimulation are relevant primarily in a pro-inflammatory microenvironment. In support, functional muscarinic receptors are expressed on the majority of inflammatory cells [5]. Also, the endogenous muscarinic receptor ligand acetylcholine and its synthesizing enzyme choline acetyltransferase (ChAT) are present in several extraneuronal cell types, including airway epithelial cells, lymphocytes, eosinophils, neutrophils, macrophages, and mast cells [5,26]. Furthermore, animal models showed that atropine reduces lung inflammation induced by diesel-soot in rats [10], and that tiotropium bromide inhibits several aspects of airway inflammation and remodeling in ovalbumin-sensitized guinea pigs, but has little effect on inflammatory cell counts in saline challenged controls [11,27]. Additionally, it has been reported that carbachol, by activation of muscarinic receptors, is able to increase inflammatory gene expression in ASM, including IL-6, IL-8 and cyclooxygenase-2 (COX-2) [28]. Furthermore, acetylcholine (ACh) can induce leukotriene B_4 _(LTB_4_) release from sputum COPD cells [4], also indicating a regulatory role for ACh in inflammatory cells. Taken together, this indicates that acetylcholine is importantly involved in the regulation of pro-inflammatory responses. Our current results provide new insights as we demonstrate that the activation of muscarinic receptors interacts with several cytokines and growth factors, in particular with TNF-α, PDGF-AB and CSE to enhance their inflammatory response in hASMc.

HASMc produce a variety inflammatory mediators [15,16,29]. This suggests an important role for ASM in inflammatory responses in COPD. Indeed, hASMc are a source of chemokines and cytokines that play a role in chronic pulmonary diseases like COPD and asthma, including IL-8 and IL-6. The levels of IL-8 are correlated with the degree of neutrophilic inflammation and are increased in sputum in COPD patients [3,30]. Several pro-inflammatory stimuli, including IL-17 [31-33], gram-positive and gram-negative bacteria [34], β-tryptase [35], IL-1β [32] and TNF-α [17] are able to induce IL-8 secretion from human ASM. Moreover, CSE synergizes with TNF-α to enhance IL-8 secretion by ASM [17]. We previously demonstrated that CSE and muscarinic M_3 _receptor stimulation leads to a synergistic increase in IL-8 secretion by hASMc [19], which as demonstrated in this study, is dependent on downstream signalling to PKC and the IκBα/NF-κB and MEK/ERK1/2 pathways. Nicotinic receptors and muscarinic M_2 _receptors are not involved in this synergism, as gallamine had no effect on IL-8 release induced by either CSE or MCh [19]. This indicates that acetylcholine may also play an important role in the inflammatory/immunomodulatory processes driven by human ASM.

Using the PKC inhibitor GF109203X, we demonstrate that the synergism of MCh and CSE-induced IL-8 secretion is mediated by PKC in hASMc. In fact, activation of PKC was sufficient to induce synergistic IL-8 secretion in combination with CSE, which was confirmed by the use of the PKC activator, PMA. These observations correspond with an earlier study from our group demonstrating that MCh augments PDGF-induced cell proliferation via the activation of PKC [23] and appear to suggest that muscarinic M_3 _receptors exert their facilitatory effects on remodeling and inflammation to an important extent via the activation of PKC. Downstream, we demonstrated that PKC is able to induce the activation of IκBα/NF-κB and MEK/ERK1/2 pathways in hASMc and that these pathways are involved in the secretion of IL-8 induced by the co-stimulation of muscarinic receptors and CSE. Interestingly, the co-stimulation with CSE and MCh appeared required to reveal the importance of PKC, as stimulation with either CSE or MCh alone was not sufficient to demonstrate an involvement of PKC. This indicates that PKC stimulation by MCh is not sufficient to induce an IL-8 or IL-6 response by itself, but augments pro-inflammatory signalling to NF-κB and ERK1/2 induced by CSE. However, synergistic functional interactions with IL-1β, an important cytokine in COPD pathogenesis [36], were not observed, both for IL-8 secretion and for activation of the signalling pathways investigated, indicating that the mechanism of the synergistic interaction is stimulus specific. Lower concentrations of IL-1β were also tested and were found to be similarly unaffected by MCh (data not shown).

The combination of MCh and CSE likely triggers PKC to activate IKK-2. This kinase allows the phosphorylation and degradation of IκBα leading to the translocation of NF-κB into the nucleus to regulate NF-κB gene transcription [37]. Furthermore, PKC has been shown to be critically involved in the activation of the ERK1/2 pathway in human aortic smooth muscle cells [38]. PKC induces the phosphorylation of Raf-1, an upstream regulator of ERK1/2 activation, which is followed by the regulation of AP-1 dependent gene transcription. The IL-8 gene contains both NF-κB and AP-1 binding sites in its promoter region [25]. Epithelial cells are also able, to induce IL-8 secretion through the activation of ERK1/2 and NF-κB in response to pro-inflammatory stimuli, including acetylcholine [8,39,40]. Taken together, these findings and our previous findings [19] indicate that the synergism between muscarinic M_3 _receptors and CSE is mediated by PKC dependent activation of the downstream pathways NF-κB and ERK1/2, to induce the secretion of IL-8.

It is unclear whether the pro-inflammatory effects of muscarinic receptor stimulation and CSE, as observed in our current work, are relevant to the COPD patient. Nonetheless, several clinical studies demonstrated that short-term therapy with tiotropium bromide improves airflow and hyperinflation [41,42]. Moreover, long-term use (up to 6 to 12 months) of this anticholinergic drug improved exercise tolerance, quality of life, rates of dyspnoea but also the exacerbation frequency in COPD patients, which are associated with periods of increased inflammatory cell influx [41,43]. The Understanding Potential Long-Term Impacts on Function with Tiotropium (UPLIFT) study concluded that COPD patients treated with tiotropium bromide during a 4-year period improved their quality of life, frequency of exacerbations and lung function, but tiotropium bromide did not reduce the decline in FEV_1 _over the treatment period [44]. Nonetheless, in a subgroup of COPD patients of the UPLIFT study, which were not on other controller medication, a reduction in the accelerated FEV_1 _decline was observed in the tiotropium bromide arm (post-hoc analysis of the UPLIFT study [44]). This was also observed in the subgroup of stage II COPD patients [45]. Collectively, besides the well described bronchodilatory effects, these findings suggest additional, non-bronchodilator properties for tiotropium bromide [6]. An anti-inflammatory role for anticholinergics is in agreement with animal and cell culture studies showing a role for acetylcholine in cell proliferation, extracellular matrix protein secretion and inflammation [5,46,47] and with our present findings showing that the inflammatory response induced by CSE, TNF-α and PDGF-AB can be augmented by muscarinic receptor stimulation in hASMc. It should be emphasized, however, that the hypothesis that tiotropium bromide may exert anti-inflammatory effects in COPD patients still needs to be tested in clinical studies.

## Conclusions

In conclusion, our results indicate that the activation of muscarinic receptors on hASMc induces the secretion of the pro-inflammatory cytokines IL-8 and IL-6, particularly in combination with inflammatory mediators and CSE. The mechanism behind the synergism between CSE- and MCh-induced IL-8 secretion involves signalling to PKC and NF-κB/ERK1/2. These and our previous findings suggest that acetylcholine might have a role in enhancing inflammatory responses.

## List of abbreviations

ASM: airway smooth muscle cells; COPD: chronic obstructive pulmonary disease; CSE: cigarette smoke extract; IKK: IκB-kinase; MCh: methacholine; PDGF: platelet growth factor; PKC: protein kinase C

## Competing interests

This study was supported by an unrestricted educational grant from Boehringer Ingelheim Pharma GmbH.

## Authors' contributions

TAO, SK, HM, PSH, SZ and RG conceived of the study and designed the experiments. AJH contributed the airway smooth muscle cell lines expressing muscarinic receptors. TAO, SK, JEN and DR performed the experiments. TAO, SK and RG analysed the data. TAO and SK drafted the manuscript. RG, HM, PSH, SZ and AJH revised the manuscript for important intellectual content. All authors read and approved the final manuscript.
